# Celiac Disease Initially Misdiagnosed as Irritable Bowel Syndrome: Case Report

**DOI:** 10.7759/cureus.71

**Published:** 2012-11-30

**Authors:** Erwa Eltayib Elmakki

**Affiliations:** 1 Department of Internal Medicine, Faculty of Medicine/ Jazan University, Jazan, SAU

**Keywords:** celiac disease, iron deficiency anemia, irritable bowel syndrome (ibs)

## Abstract

Background: The increasing availability of serological testing & upper endoscopy has led to more frequent diagnosis of celiac disease & recognition that it may mimic Irritable bowel syndrome (IBS).

Objective: The objective of the present case report is to describe the importance of screening those with vague abdominal symptoms (like patients with IBS) and iron deficiency anemia for celiac disease.

Methods: We report the clinical course of a 30-year-old patient with vague abdominal symptoms initially misdiagnosed as having IBS; when the patient presented in our clinic, he was noted to have iron-deficiency anemia. On work-up for the cause of iron deficiency anemia, he was found to have celiac disease on basis of positive serological tests and small bowel biopsy result. After being placed on gluten-free diet, plus iron supplements, his abdominal symptoms and iron deficiency anemia totally improved.

Conclusions: Our case demonstrates that routine screening for celiac disease should highly be considered for patients with iron-deficiency anemia and IBS.

## Introduction

Celiac disease or gluten sensitive enteropathy is an intolerance of dietary gluten that results in immunologically-mediated inflammatory damage to the small intestinal mucosal. The damage is characterized by inflammation, crypt hyperplasia, and villous atrophy [1].

## Case presentation

**Figure 1:**
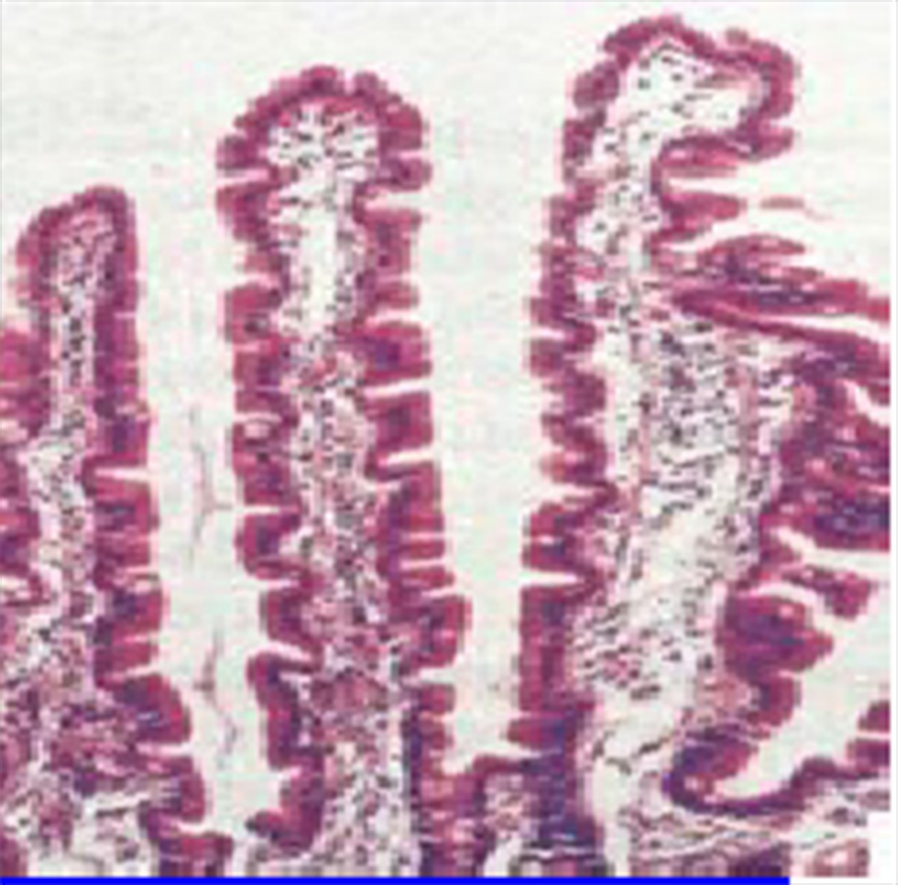
Normal mucosal histology PeerEmed Legacy Image

**Figure 2:**
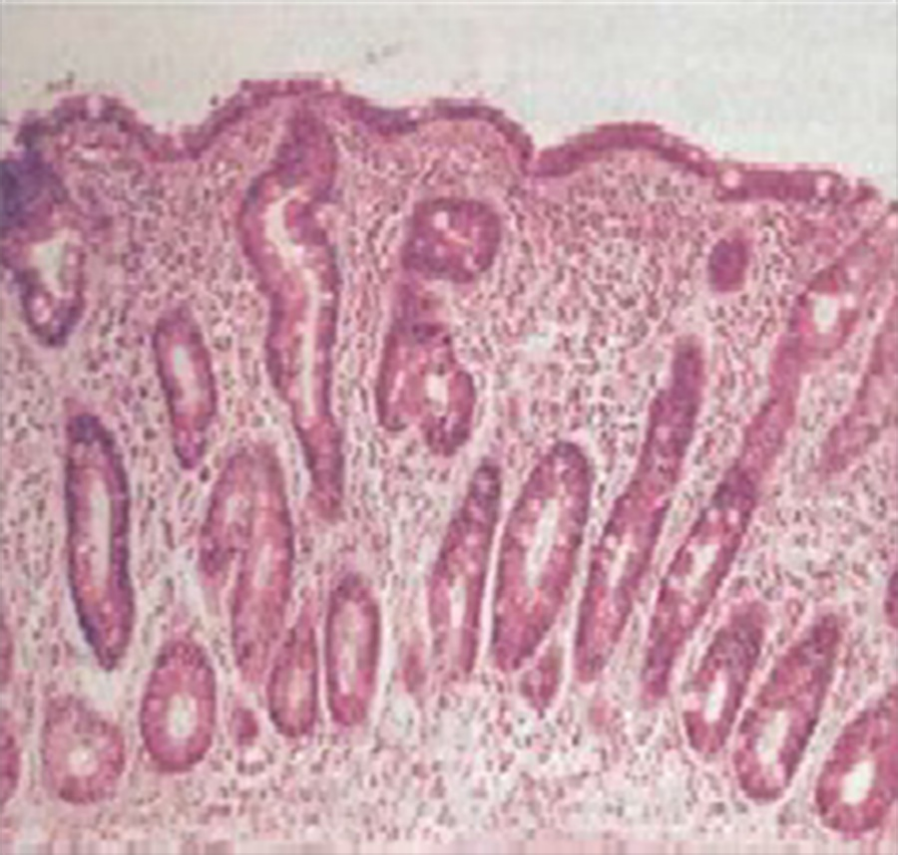
Subtotal villous atrophy PeerEmed Legacy Image

A 30-year-old Saudi male teacher was referred to our clinic because he was incidentally found to be positive for HBsAg. For the last 18 months prior to the date of referral to our clinic, he had been treated as having IBS in another GI clinic, diagnosis of IBS was made on clinical basis as he gave a history of recurrent episodes of abdominal bloating, vague abdominal pains and diarrhea, alternating with constipation. Otherwise, his appetite was good. There was some weight loss but no history of gastrointestinal bleeding. He had neither extra-intestinal symptoms nor past medical history of note.  Physical examination revealed no abnormalities. Routine work-up for HBV showed that he was an inactive carrier with normal liver enzymes, very low viral load, -ve HBeAg, and +ve HBeAb; however, his CBC revealed microcytic hypochromic blood film with low hemoglobin (9.3gm/dl), so further work-up results were as follows:

Low serum iron & ferritin, endoscopic small bowel biopsy showed subtotal villous atrophy, plus inflammatory cells infiltrate (Figures 1, 2), positive anti-tissue transglutaminase (IgA) with a high level (126), positive anti-endomysial antibodies, low serum albumin and calcium, high PT and PTT, high alkaline phosphatse, low triglyceride with normal serum cholesterol, high TSH with normal T3 and T4, normal LFTs and RFTs, normal serum B12 and folate, and a normal abdominal ultrasonography and abdominal CT scan with IV contrast. Finally, a diagnosis of celiac disease was made, and the patient was put on a gluten-free diet, in addition to ferrous sulphate 200 mg BID and calcium supplement. Two months later, the patient’s abdominal symptoms were dramatically improved and Hb was 12 gm/dl.

## Discussion

Celiac disease can present at any age. The peak incidence in adults is typically in the fifth decade with a female preponderance. The symptoms are very variable and often non-specific with tiredness and malaise often associated with anemia. Many patients are asymp­tomatic (silent) and picked up on incidental findings. Common GI symptoms of celiac disease include diarrhea or steatorrhea, abdominal discomfort, bloating or pain, and weight loss. Mouth ulcers and angular stomatitis are frequent and can be intermittent. Infertility and neuro-psychiatric symptoms of anxiety and depression also can occur [2].

Patients with IBS suffer from biopsy-proven celiac disease at rates that are more than four times higher than in non-IBS control subjects. This was the conclusion of a systematic review and meta-analysis conducted by Alexander and his colleagues [3]. 

Also, it is recommended to screen for celiac disease in patients with newly diagnosed chronic fatigue syndrome, IBS and autoimmune thyroid disease [4-6]. Some authors have recommended that patients with iron deficiency anemia be routinely assessed for celiac disease [7]. 

In our case, his clinical symptoms were initially suggestive of IBS, and because our current medical guidelines do not always call for celiac screening in these individuals, the diagnosis was initially missed. However, with the progression of the disease, he developed weight loss and features of iron deficiency anemia, and these signs led us to screen him for celiac disease. Enomysial (EMA) and tissue transglutaminase IgA (TGA) are the most sensitive and specific antibodies for the diagnosis of untreated celiac disease and can also be used as screening tests. Treatment of celiac disease with gluten-free diet usually produces a rapid clinical and histological improvement. In untreated cases, the incidence of enteropathy-associated T-cell lymphoma (EATCL) is increased [2].

In our case, both endomysial and tissue transglutminase antibodies were positive; but for more confirmation, we obtained endoscopic biopsy from the third part of the duodenum which was in keeping with celiac disease (showing subtotal villous atrophy), and on top of that, our patient responded to gluten-free diet. So screening patients with vague abdominal symptoms and unexplained iron deficiency anemia for celiac disease by serological tests is cost effective as treatment of celiac disease is simple, and at the same time, if diagnosis of celiac disease is missed, the patient will be at risk of developing small bowel malignancy.

One of the abnormal laboratory findings in our case is the elevation of TSH with normal T3, T4; these thyroid dysfunctions are consistent with subclinical hypothyroidism. It has been reported that CD can be seen in association with thyroid dysfunction [8].

## Conclusions

This case demonstrates the significance of screening patients with vague gastrointestinal symptoms for celiac disease, at least using serological tests, as celiac disease is a treatable condition.  
